# The construction of elastin-like polypeptides and their applications in drug delivery system and tissue repair

**DOI:** 10.1186/s12951-023-02184-8

**Published:** 2023-11-11

**Authors:** Yingshu Guo, Shiwei Liu, Dan Jing, Nianzu Liu, Xiliang Luo

**Affiliations:** 1 School of Chemistry and Chemical Engineering, Qilu University of Technology (Shandong Academy of Sciences), Jinan, 250353 China; 2 Key Laboratory of Optic-Electric Sensing and Analytical Chemistry for Life Science, MOE, College of Chemistry and Molecular Engineering, Qingdao University of Science and Technology, Qingdao, 266042 China

**Keywords:** Elastin-like polypeptide, Biomaterial, Drug delivery, Tissue repair

## Abstract

Elastin-like polypeptides (ELPs) are thermally responsive biopolymers derived from natural elastin. These peptides have a low critical solution temperature phase behavior and can be used to prepare stimuli-responsive biomaterials. Through genetic engineering, biomaterials prepared from ELPs can have unique and customizable properties. By adjusting the amino acid sequence and length of ELPs, nanostructures, such as micelles and nanofibers, can be formed. Correspondingly, ELPs have been used for improving the stability and prolonging drug-release time. Furthermore, ELPs have widespread use in tissue repair due to their biocompatibility and biodegradability. Here, this review summarizes the basic property composition of ELPs and the methods for modulating their phase transition properties, discusses the application of drug delivery system and tissue repair and clarifies the current challenges and future directions of ELPs in applications.

## Introduction

Biomaterials are frequently employed in medicine to achieve therapeutic or diagnostic goals [1, 2]. These biomaterials, which may be natural or synthetic, often contain degradable components [3]. The biological properties of the natural proteins are maintained, but the design can be tailored to the application scenario. [4]. The extracellular matrix (ECM) is rich in natural elastin. Since the discovery of the Val-Pro-Gly-Val-Gly repeat amino acid sequence in natural elastin by Urry et al. in 1973 [5], researchers have used genetic engineering to generate elastin-like polypeptides (ELPs) that inherits the biocompatibility and elastic retraction capabilities of natural elastin. Accordingly, these ELPs are believed to be highly suitable for simulating the extracellular environment of elastic tissues [6].

**Fig. 1:**
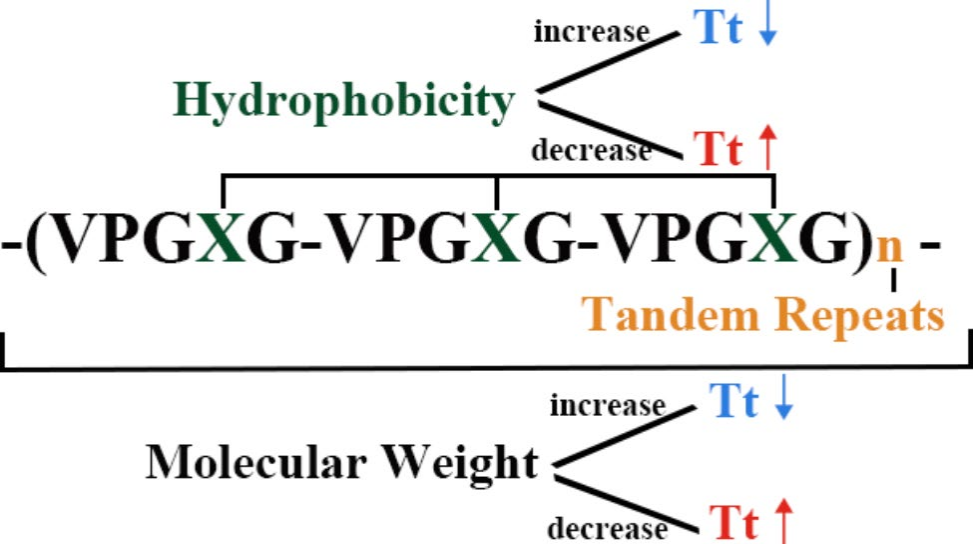
The structure of ELP and its impact on its own Tt

Since Urry’s work in the 1970s, researchers have been examining the physicochemical features of ELP and the polypeptide motif that supports its structure [7–9]. The pentapeptide Val-Pro-Gly-X-Gly, abbreviated as (VPGXG)_n_, makes up the majority of ELPs, wherein X denotes an amino acid that is interchangeable. (Fig. 1) However, Proline is the only amino acid for which the X cannot be adopted. Moreover, the characteristics of ELPs can be modified by changing either X or n [6]. Thus, by altering the sequence composition, it is possible to achieve precise modulation of the physicochemical and biological properties of the biomaterials to better meet the needs of biological applications.

Similar to poly (N-Isopropyl acrylamide), ELPs are synthetic thermosensitive polypeptides, and the length of their polypeptide chains and guest groups have a key influence on their thermal response properties [10, 11]. Accordingly, the phase transition temperature (Tt) of ELPs can be rationally regulated to be close to the physiological temperature by adjusting their sequence composition, peptide chain length, and concentration [12]. Below a specific phase-transition temperature, ELPs typically adopt a disordered, more hydrophilic structure, dissolve well in aqueous solutions, and exist as monomers. This behavior is known as lower critical solution temperature (LCST). In the presence of phase separation, conversely, ELPs take on a dynamic, regular, nonrandom structure called a β-spiral above Tt, thereby losing its water molecules, undergoing physical property changes [13] and condensing into a portion [14]. This phenomenon is defined as the inverse temperature transition (ITT) [15, 16].

The process of adding amino acids from the C-terminus to the N-terminus consecutively is known as solid-phase peptide synthesis, and it is frequently used to synthesize ELPs with reduced molecular weight [17]. This includes the Boc synthesis method, which uses benzyl alcohol as the side chain protecting group and Boc as the α-amino protecting group. In addition to the above, Fmoc synthesis techniques that use Fmoc as the α-amino protecting group have also been reported [18].

Although the chemical synthesis method is efficient and allows the addition of unnatural amino acids, it has the disadvantages of high synthesis cost, poor schedule, and difficulty in mass production. Thus, using genetic engineering approaches to generate ELPs is a viable option at present [19].

The chemical composition of engineered peptides can be similar to complex natural proteins, and they can be freely altered at the genetic level to generate entirely synthetic proteins. In this regard, *Escherichia coli* (*E. coli*) has been frequently used to synthesize recombinant proteins and peptides because it is the most cost-effective host for recombinant expression (cheaper than yeast or mammalian production). [20–22] Using genetic engineering, the length and functional groups of guest residues are modified, and functional peptide motifs are incorporated to interact with other molecular species or the environment to establish the desired functions. The plasmid containing the ELP sequence is then transferred into the bacteria, and the ELPs are extracted from the bacterial samples [23, 24]. This method has several advantages. The well-defined amino acid sequence can facilitate the modulation of the physical and chemical properties of ELPs [25]. Secondly, studies have shown that post-translational modifications (PTMs) can further enrich the functions of ELPs. For example, a 11-amino acid peptide from the natural myristoylated yeast protein Arf2 was used as a substrate, and the Arf2 recognition sequence was fused to the N-terminus of ELP at the gene level, and expressed in BL21(DE3) E. coli. After culturing for a period of time, exogenous myristic acid and isopropyl β-D-thiogalactoside (IPTG) were added to the medium, and then myristoylated ELP was obtained. [26] Mozhdehi et al. incorporated natural lipidation PTM (hedgehog-mediated cholesterol modification of proteins) into the ELP sequence, and obtained cholesterol-modified ELPs by adding cholesterol to the solution after expressing them in E. coli. [27] Furthermore, this method is easy to operate and the reagents used in the process can effectively reduce the environmental pollution.

Inverse Transition Cycling (ITC) is a chromatography-free purification method that relies on the LCST properties of ELPs. ITC has been widely used for the purification of ELPs [15–18, 25, 19]. Using the thermoresponsive characteristics of ELPs, Meyer and Chilkoti et al. created the inverse conversion cycle (ITC) for the purification of ELP-labled soluble fusion proteins. [28]. The addition of soluble salts to the ELP solution or the adjustment of the appropriate temperature enables the effective separation and purification of ELP-labeled proteins, and the adjustment of the ELP amino acid structure creates a suitable separation environment for different proteins. After phase separation, impurities are present in the supernatant and the ELP-labeled target protein becomes precipitated. This process is quick, simple, and inexpensive. [29, 30].

As a thermoresponsive peptide, ELPs can self-assemble into different nanostructures by adjusting the composition of the peptide chain structure, including micelles [31–33], vesicles [25, 34–36] and nanofibers [37, 38].

ELPs are particularly appealing as biomimetic polypeptides owing to their excellent properties, such as conserved sequence, biocompatibility, stimuli-responsive behavior, and lack of intrinsic cytotoxicity [14–17]. Accordingly, ELPs have been investigated for regulating Tt in drug delivery systems as well as for targeting cells using a temperature response mechanism [12]. Thus, ELPs can be used for medical and biotechnological applications, making them versatile building components [39]. However, due to their high Tt, short synthetic ELPs (less than 10 pentapeptides) have not been utilized in thermos-responsive drug delivery systems [40].

**Fig. 2:**
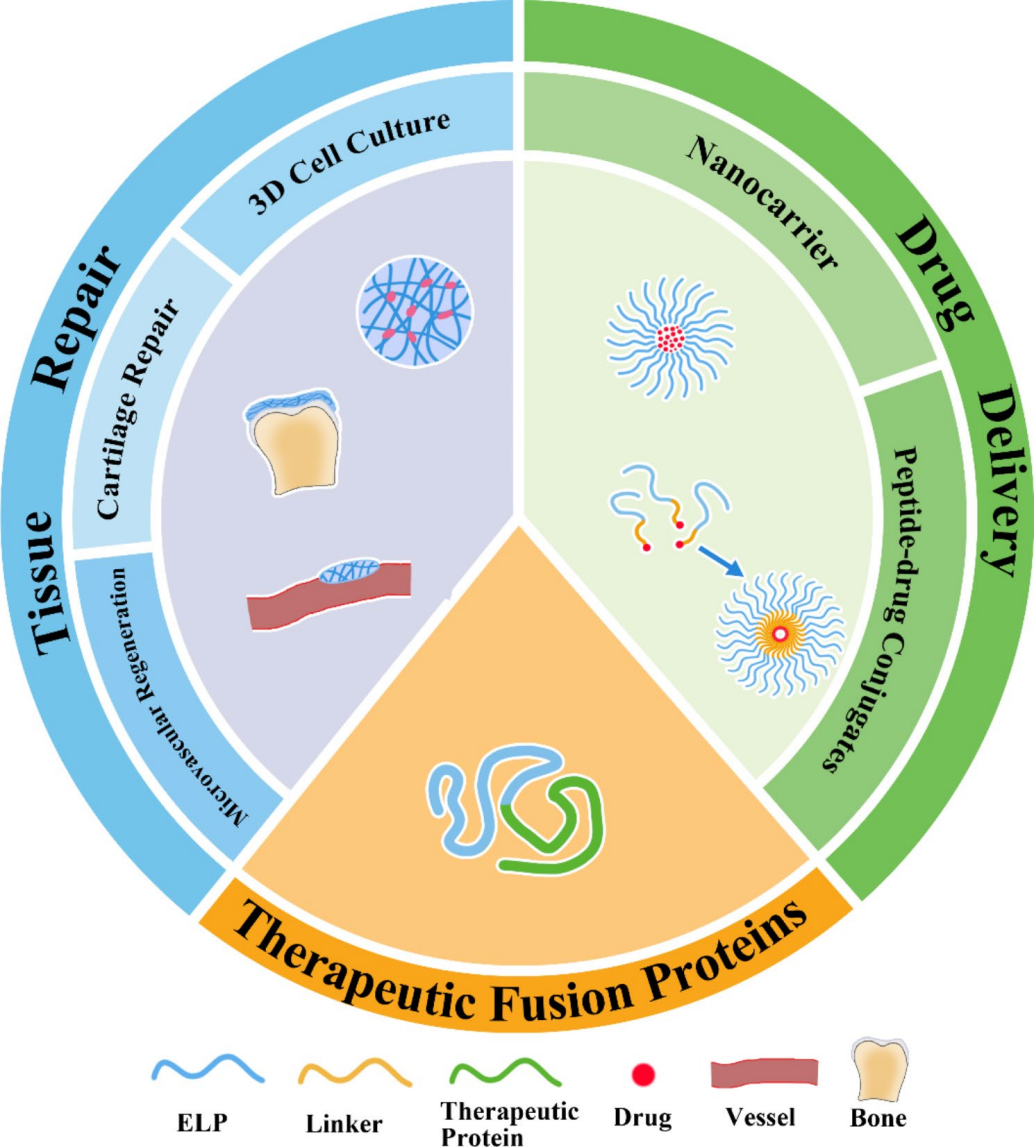
Customizable ELP applications in drug delivery system and tissue repair

Due to the fact that ELP sequences are derived from natural elastin proteins, they have low immunogenicity, are biodegradable, and can enhance pharmacokinetics through sequence structure control [25, 29, 30], so they are widely applied in the field of drug delivery and tissue repair. (Fig. 2). Their low platelet adhesion and immunogenicity make them excellent for use as drug carriers, and their inherent stimulus responsiveness to self-assemble into various nanostructures makes them a hot topic in stimulus-responsive drug carrier research [41, 42]. In this regard, by controlling the zeta potential and particle size, ELPs biomaterials have been widely used in disease treatment research. [14, 43, 44].

Artificially synthesized ELPs exhibit excellent biocompatibility with tissue microenvironments and find extensive applications in tissue engineering [45, 46]. ELP retains its thermal responsiveness characteristics even after fusion with other peptides [28]. Most studies show that before therapeutic protein fusion, ELP needs to be sequence adjusted in advance to reduce the impact on the activity of the therapeutic protein [47–62]. Improved pharmacokinetics and improved bioavailability can then be achieved. Overall, these features indicated how ELP has considerable potential as customizable platforms for drug delivery system and tissue repair.

## Construction methods for ELPs

In 1992, the Urry laboratory [63] achieved the expression of ELP(V)_20_ for the first time through Escherichia coli, by inserting the ELP(V)_10_ gene into the vector via a single enzyme cut. Due to the limitations of single enzyme cutting techniques, synthesizing ELPs with a large number of repeat sequences proved challenging. To address this issue, the Urry laboratory [64, 65] constructed ELP(V)_121_ using a concatenation ligation reaction, successfully expressing it within the E. coli system. This reaction, which has a shorter duration, allowed for the connection of ELP monomer genes through overlapping sticky ends, resulting in genes with multiple repeat sequences. However, this method did not provide precise control over ELP length. In 2002, the Chilkoti laboratory [66] developed the recursive directional ligation (RDL) technique, which involved multi-step single enzyme cuts to insert ELP monomer genes or oligomer genes into linearized vectors, enabling precise synthesis of ELPs with specific sequence lengths.

Furthermore, due to its modular nature, the RDL method allowed for fusion of ELPs with other proteins, with the fusion sequence freely definable. Building upon RDL, the Chilkoti laboratory [67] introduced a second enzyme cutting site, further advancing the method into plasmid reconstruction-based recursive directional ligation (pre-RDL). This method effectively overcame the limitations of RDL and has become the most commonly used approach for constructing ELPs.

Simultaneously, the Chilkoti laboratory [19] developed another method for synthesizing ELP genes, a rapid, high-throughput, one-step approach known as overlap extension rolling circle amplification (OERCA). This method utilized circular ssDNA as a template to generate a multitude of linear ssDNA fragments of varying lengths, which were subsequently used as templates for polymerase chain reactions (PCR) to extend gene length. OERCA is a potent technique suitable for constructing ELP libraries with varying sequence lengths, but it is limited to synthesizing ELPs with relatively simple sequence compositions and cannot handle complex sequences. In 2016, the Chilkoti laboratory [68] proposed a new method that leveraged codon degeneracy to optimize ELP genes using a codon scrambling approach. This method employed gene-specific primers for PCR amplification, reducing the occurrence of cross-hybridization or off-target reactions during the polymerase reaction. This approach significantly addressed the challenge of synthesizing highly repetitive encoded sequences and is poised to become a novel method for constructing ELPs.

## Factors affecting ELP phase-transition temperature

By modifying the peptide sequence, it is also possible to further modify the ELP’s Tt to particular physiological conditions. The introduction of hydrophilic amino acids raises the ELP’s Tt, while the incorporation of amino acids with hydrophobic side chains lowers the Tt. When X carries a charge, the Tt of ELP increases, and when X does not carry a charge, the Tt of ELP decreases [13]. Additionally, the Tt of ELP is affected by the number of repeating units in a protein, how they are constructed, how they are arranged, how they are dissolved in a solution, and if they are conjugated with other biomolecules [14, 18]. Different structures of ELPs were made by genetic engineering, and these ELPs have different Tts due to different amino acid sequence structures. To further meet the application conditions, chemical modification of the structures of the existing ELPs is a common approach and the use of orthogonal chemoselective bioconjugation reactions to obtain multiple ELP derivatives with different thermoresponsive behavior [69].

### Effect of ELP side chain modifications on Tt

Post-translational modifications enhance the functionality of ELPs by allowing the modification of protein structure and function in diverse biological environments. Mozhdehi et al. prepared ELPs modified by three fatty acids through recombinant expression and post-translational lipidation methods. These fatty acid-modified ELPs are composed of an amphipathic domain, a post-translationally C_14_ alkylated β-sheet-forming peptide fused to a thermosensitive elastin-like polypeptide. They exhibit temperature-triggered hierarchical self-assembly at multiple length scales, with distinct structural and material properties that can be controlled at the sequence level. This shows the feasibility of using post-translational modification to modify the structure of ELPs. [70] Correspondingly, ELPs have been modified synthetically by selectively modifying the amino acid side chain or terminal modifications. In addition, double orthogonal reactions have been and are still widely used, in addition to numerous chemical methods that ELPs can be selectively modified with cysteine, lysine, tyrosine or tryptophan on their backbone, thus enriching the functions of ELPs [71].

Binding engineered aminoacyl RNA synthetases to noncanonical amino acids (NCAAs) has also surfaced as a powerful approach to expand protein chemistry libraries [72]. Amiram et al. investigated the effect of various nonnatural aromatic moieties on the LCST phase transition behavior of ELPs. The incorporation of aromatic NCAAs resulted in a variety of ELPs with a wide range of LCST behavior (across 60 °C). In addition, the LCST behavior of ELPs gradually reduced as the hydrophobicity of aromatic NCAAs increased [73]. Chilkoti et al. designed two types of ELPs with photocrosslinked p-azidophenylalanine (pAzF) residues: photocrosslinked ELP (PCE) and self-assembled photocrosslinkable diblock ELP (PCD). As evident from the results of the bulk LCST phase separation of PCE, the concentration-dependent transition temperature also known as cloud points of PCE decreased at each concentration compared to noncrosslinkable ELP (NCE) due to the presence of four pAzF residues along the polypeptide backbone. Moreover, pAzF residues do not adversely affect the LCST behavior of PCE [74]. Thus, when a predetermined amount of azobenzene is added to a specific location of the ELP, the Tt of ELP decreases with a corresponding increase in the azobenzene concentration. Correspondingly, azobenzene-modified ELPs are competent to achieve soluble to insoluble phase transitions under isothermal reversible conditions under light exposure, and the Tt of ELPs during cis-trans azobenzene isomerization differ by up to 12 °C [8].

In order to control Tt by changing X, the peptide chain structure of ELPs should be constantly adjusted, and suitable amino acids should be selected for modification, so that Tt can meet the expected requirements. Accordingly, Lim et al. developed a peptide-based platform for developing temperature-responsive and bioactive ELPs. In the final step of peptide synthesis, a single amino acid was changed at the N-terminus to accord a facile modification of the critical temperature affecting the thermoresponsive behavior. Moreover, the Tt of ELPs is also regulated by hydrophobicity. As hydrophobicity increases, Tt decreases. [75].

**Fig. 3:**
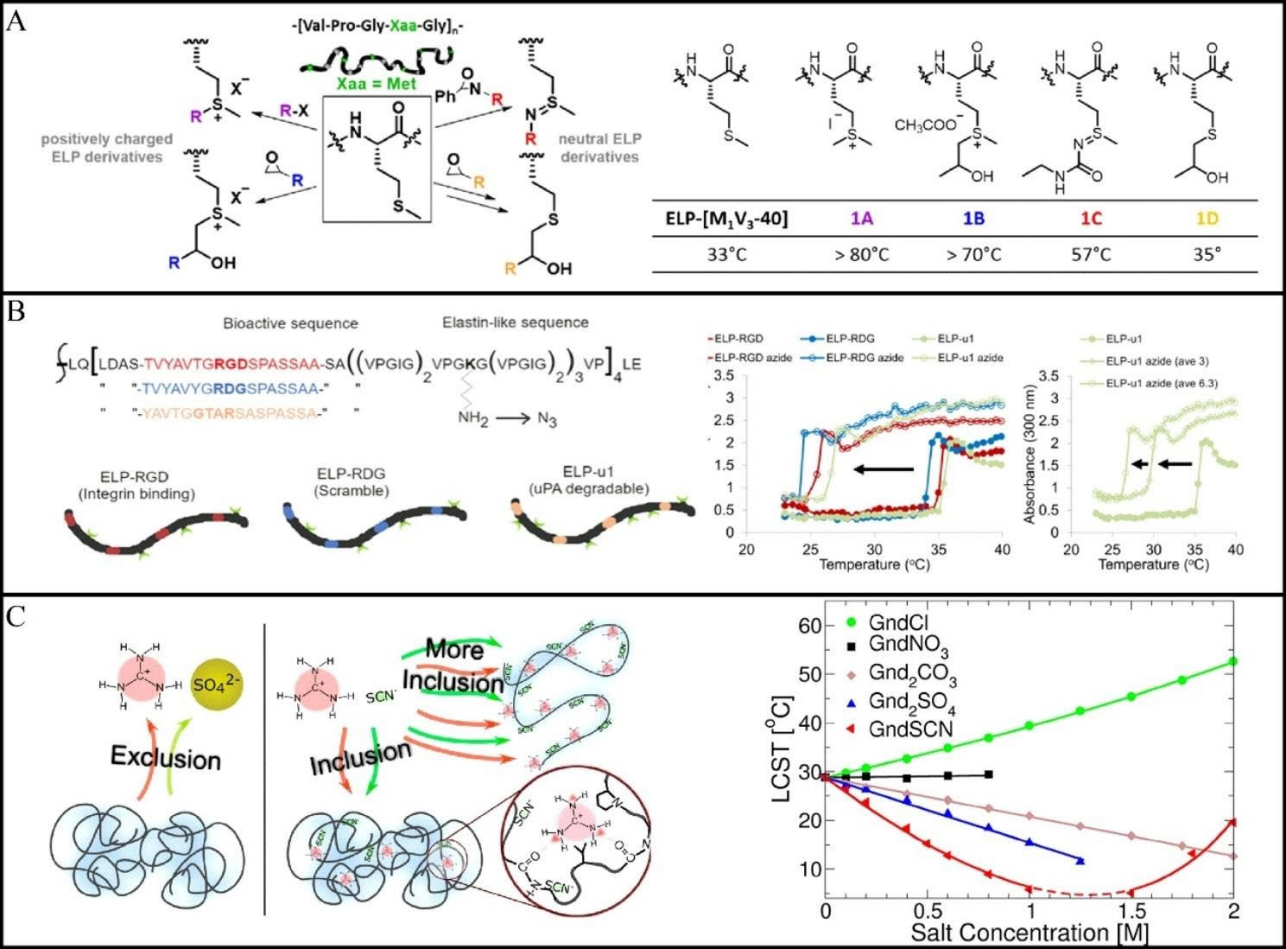
** A** The chemical structure of ELP with alanine side chain modifications and their respective Tt. (Reproduced with permission from Ref. [71], Copyright 2019, American Chemical Society) **B** The LCST of all ELP sequences is decreased due to azide group modification. The shift in LCST depends on the number of primary amines converted to the azide group. (Reproduced with permission from Ref. [78], Copyright 2020, American Chemical Society) **C** GndSCN contains guanidine and thiocyanates that bind to the peptide skeleton. LCST measurement of 10 mg/mL ELP solution versus guanidinium salt concentration. (Reproduced with permission from Ref. [82], Copyright 2017, American Chemical Society)

Chemoselective modifications at the position of the guest residue, particularly side chain alkylation of methionine-containing ELPs, can also be used to modify the characteristics of ELPs [76]. Under acidic conditions, the thioether group is able to bioalkylate with the methionine side chain at a lower pH. Based on ELP[M_1_V_3_-40], Lecommandoux et al. studied a variety of methionine modifications to modulate the thermoresponsive characteristics of ELPs. Correspondingly, methionine-containing ELPs were modified in a chemoselective manner using oxazine-based reagents. According to the findings, the Tt values of ELP[M_1_V_3_-40] in the concentration range of 25–250 µM were found to range from 30 to 37 °C, and the thioalkyl derivatives did not exhibit notable thermal response characteristics in the examined temperature and concentration ranges. In addition, the increase in hydrophilicity and solubility was attributed to the sulfonium groups in the polypeptide. Accordingly, the higher Tts (54–69 °C) of the compound at concentrations between 25 and 250 µM were attributed to the polar sulfonimide bond having similar characteristics to the sulfoxide bond (its oxygen analog) (Fig. 3A) [71].

In addition to methionine alkylation, various amino acids have been alkylated to tune the thermal response behavior of ELPs [77]. In this regard, Lampe et al. used a diazo transfer reaction to modify the primary amine of lysine on each ELP sequence with an azide. Because anodizing lysine residues on the ELP is equivalent. When the lysine residues on the ELPs chain are modified by azide, the increase of hydrophobicity leads to the decrease of Tt, and the more azide there is the more the decrease of Tt. (Fig. 3B) [78].

### The effect of environmental media on Tt

Factors such as salt ions, pH, charge density, organic solvents and external pressure in the solvent environment will also affect the phase transition temperature of ELPs [79, 80], and in subsequent studies researchers have explored the effects of inorganic salts, charge density, and organic macromolecules in solution on Tt [81, 82].

Adding sodium salt to an ELP solution causes the LCST to conform to the Hofmeister effect, moreover, the addition of NaCl has been demonstrated to effectively lower the LCST of ELP [83]. In this regard, Liu et al. evaluated the LCST of ELY_16_ and mELY_16_ under conditions relevant to biomedical applications, Accordingly, the LCST were reduced by adding salt to PBS or increasing the peptide concentration; the LCST was reduced to 26 °C when salt was added to 150 mg/mL of PBS [84]. In addition, Pirzer et al. used ELPs with stimulus-responsive characteristics to regularly alter the shape of rectangular DNA origami structures, they found that the change in temperature is independent of the rectangle concentration; however, the Tt can be systematically changed by adding 0.5 M NaCl, 1.0 M NaCl, or 1.5 M NaCl to PBS and varying the NaCl concentration. In addition, Tt was higher than 55 °C when NaCl ≤ 0.5 M was added, while Tt decreased to below 10 °C when NaCl ≥ 2 M [85].

Additionally, Cremer et al. uncovered a unique mechanism based on the accumulation of Gnd^+^ and the counteranion into the collapsed form of the macromolecule at low concentrations of GndSCN. Gnd^+^ pairs with the hydrated anion in solution, which in turn stabilizes the collapsed state of ELP. At lower salt concentrations, pairing of Gnd^+^ with anions was also found to cause aggregation of ELP chains. While at higher salt concentrations, Gnd^+^ was distributed with SCN^-^ on the polymer surface, preventing the aggregation of ELPs (Fig. 3C) [82].

However, the interaction mechanism between aqueous solutions and ELPs has been studied more, but there is less research on the interaction between organic macromolecules and ELPs. [75, 78] As a crowding agent, Polyethylene glycol (PEG) could influence the phase transition behavior of ELP in single salt solutions. Zhang et al. studied the effect of PEG on the phase transition of SpyCathcher–ELPs_40_ (E–C) in the presence of different salts. Tt_E-C_ decreased proportionally with increasing PEG concentrations when the concentration of Na_2_CO_3_ was below 0.5 mol/L [86].

## Application in drug delivery system and tissue repair

As a synthetic polypeptide, ELPs have excellent biocompatibility and are widely used to generate novel therapeutic proteins with therapeutic polypeptides, tissue repair and drug delivery [39, 46, 87]. Using genetic engineering technology and bio-orthogonal click reaction, it is possible to assemble structures such as drugs, unnatural amino acids, and chemically modified functional peptides onto the ELPs chain [24]. Moreover, changing the sequence via genetic engineering enables the binding of therapeutic proteins to ELPs to form new fusion proteins that retain the therapeutic properties of the therapeutic protein while also possessing ELP biocompatibility and temperature-responsive behavior (Table 1). The sequence of ELP is precisely controllable, and through genetic engineering to design site-specific coupling reactive residues, covalent coupling of ELP to small molecule drugs can be achieved. [88]

**Table 1:** Therapeutic fusion proteins based on ELPs discussed in this review

Proteins composition	Transition temperature (Tt)/℃	Half-life (t_1/2_)/h	Bioactive part	Therapeutic effect	Ref
IFN-ELP_90_	45.3	8.6	IFN	Inhibit tumor growth. Prolong the survival time of mice. No hemolysis.	[48]
IFN-ELP(V)_90_	37	280 ± 0.5	IFN	Stimulate anti-tumor immune response, Inhibit the recurrence of glioblastoma.	[49]
IFN-α-MMPs-ELP(V)_90_	< 37	422.2 ± 13.7	IFN-α	Improve anti-tumor efficacy. Increase intratumoral accumulation.	[50]
α-FLT3-ELP(A) _192_	42.3	14.7	α-FLT3	The fusion protein has high stability and specificity, Effective therapeutic effect on AML.	[51]
α-CD99-ELP(A)_192_	45.3	15.8	α-CD99	Reduces cell viability of AML cell lines, Reduced leukemia burden in mice.	[52]
DRA-ELP(V)_120_	25	-	DRA	Eliminate DRA-sensitive tumor tissue	[55]
mini cry -ELP(S)_48_(I)_48_	30	2.8	mini cry	Inhibit RPE apoptosis and caspase-3 activation and protect the retina from cell death.	[57]
FGF-21-ELP_120_	~ 30	16.6 ± 3.9	FGF-21	A single injection can control blood sugar for 5 days.	[59]
vRAGE-ELP(V_40_C_2_)	30	-	vRAGE	Reduce expression of pro-inflammatory factors, Accelerate wound healing in mice.	[61]

### Therapeutic fusion proteins based on ELPs

Therapeutic proteins have received considerable attention for treating numerous diseases. Peptides comprise desirable chemotherapeutic carriers because of their low toxicity, recombinant production, and ability to self-assemble into nanoparticles that enhance drug pharmacokinetics [87]. Using genetically encoded targeting ligands, peptide-based systems can achieve active drug targeting. This offers a new degree of control and increases treatment efficacy, whereas passive targeting can facilitate the accumulation of nanocarriers in solid tumors [46]. However, there is rapid renal clearance of therapeutic proteins as well as rapid protein hydrolysis, which often requires high dose administration and leads to adverse effects. [89] By fusing with other proteins and peptides at the N-terminal or C-terminal end, ELPs can form new therapeutic proteins with various therapeutically active proteins. For example, after fusing the antibody of tumor necrosis factor (TNF) with ELP, the molecular weight of the fusion protein is larger and it will not be filtered out by the glomerulus. Its half-life in the body is extended from 28 min to 11.4 h compared to the TNF antibody without ELP fusion. [90] Under physiological conditions, due to the thermoresponsive nature of ELPs, they can often form a therapeutic protein reservoir, slowly produce therapeutic effects, and increase the half-life of therapeutic proteins. [60, 91].

To overcome the vulnerability of peptide and therapeutic protein drugs to short half-lives and immunogenicity, Isaacs et al. doped ELP with multiple pAzFs, which have programmable binding affinity for albumin and maintain fusion protein activity, with adjustable serum half-lives in mouse models ranging from 5 to 94% of the albumin half-life, they prepared a thioredoxin (Trx)-ELP fusion protein and evaluated the activity of the recombinantly produced Trx, Trx-ELP (10pAzF), and Trx-ELP (10FA). The biological activity of Trx and Trx-ELP (10pAzF) was similar. However, after treatment with an excess of human serum albumin (HSA), the activity of trx-ELP (10FA) decreased by 50% due to HSA binding, while the activity of Trx and Trx-ELP (10pAzF) did not significantly decrease [47].

**Fig. 4:**
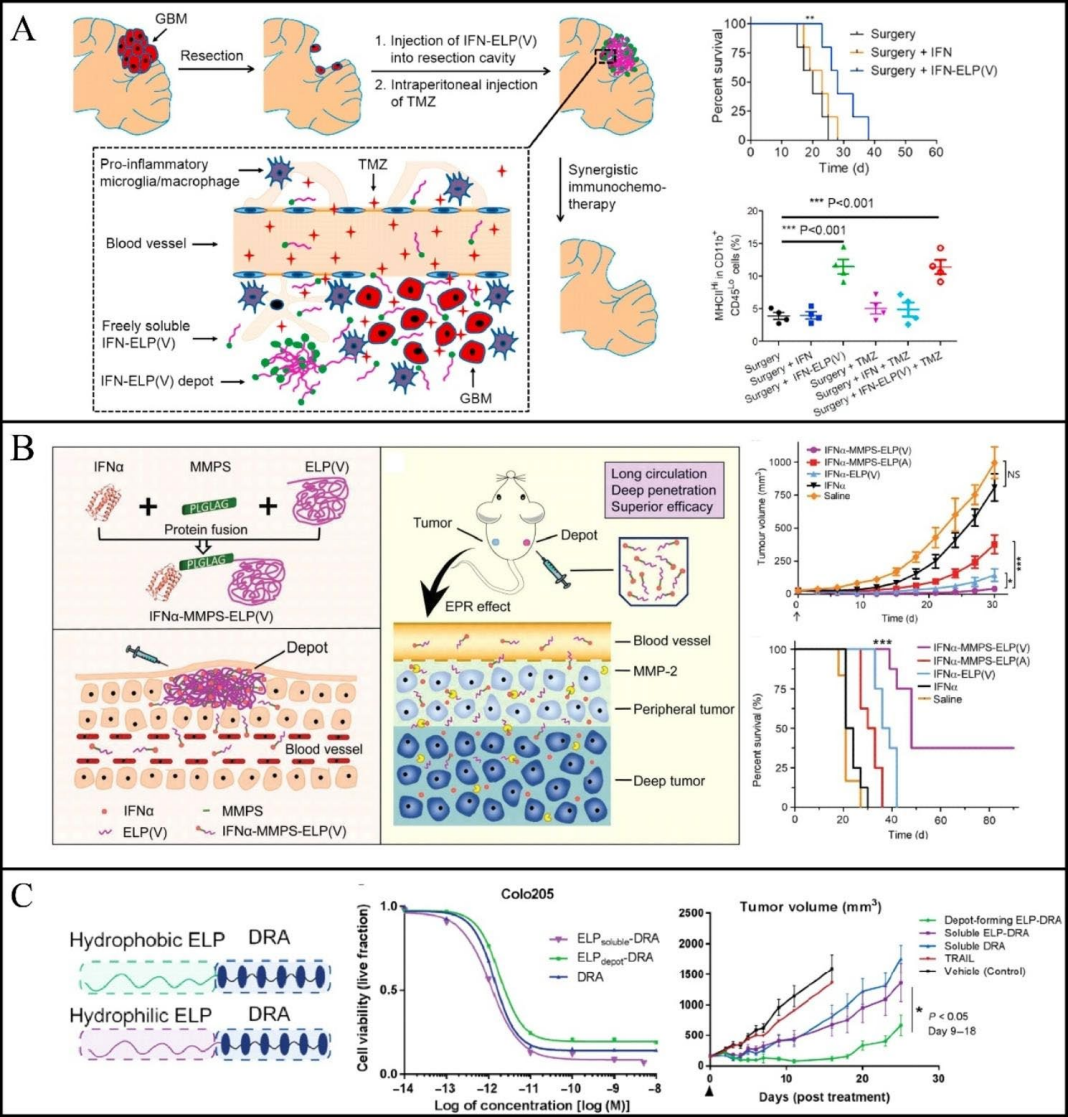
** A** IFN-ELP(V) and TMZ achieved synergistic treatment after GBM. Cumulative survival of mice after IFN-ELP(V) injection. Local anti-GBM immune response triggered by IFN-ELP(V). (Reproduced with permission from Ref. [49], Copyright 2020, Elsevier Ltd) **B** Design scheme of IFNa-MMPS-ELP(V) Subcutaneous depot formation after IFNa-MMPS-ELP(V) injection. In vivo therapeutic mechanism of IFNa-MMPS-ELP(V) Melanoma growth inhibition. Survival curve of animals with ovarian cancer. (Reproduced with permission from Ref. [50] Copyright 2019, The Authors) **C** Composition of ELP_depot_-DRA and soluble controls. Different forms of ELP-DRA have similar cytotoxicity to Colo205 as DRA. There was a significant difference in tumor volume between ELP_depot_-DRA at day 9 and day 18. (Reproduced with permission from Ref. [55] Copyright 2019, The Authors)

Relevant clinical activity of interferon-α (IFN-α) has been attributed to its immunostimulatory properties, although it was once believed to inhibit tumour activity by activating IFN-α tumour-associated receptors. To address the issues of short IFN half-life and frequent dosage, Gao et al. fused the C-terminus of IFN-α with ELP to generate the IFN-ELP fusion protein, known as IFN-α-ELP. Because the C-termi nus of IFN-α is located far from the identified receptor binding domains, they believed that ELP modification at the C-terminus of IFN-α would not significantly affect its biological activity. When compared to free IFN-α, the fusion protein retained IFN’s in vitro biological activity, the half-life of circulation was extended nearly 30-fold, and tumor accumulation also significantly increased [48]. In their subsequent work, the therapeutic efficacy of this IFN-α-ELP fusion protein in postsurgical glioblastoma (GBM) immunochemotherapy was re-evaluated. IFN-α-ELP(V) can be slowly released from the drug reservoir, exhibits zero-order release kinetics, considerably improves pharmacokinetics and biodistribution, penetrates surrounding brain tissue, stimulates local antitumor immune responses, and can prevent recurrence of GBM (Fig. 4A) [49].

At physiological temperature, IFN-α-MMP substrate (MMPS)-ELP designed by Gao et al. was able to form a drug reservoir after subcutaneous injection, which in turn resulted in a slow release into the physiological circulation, significantly improving the pharmacokinetics [50]. Matrix metalloproteinases (MMP) are overexpressed in tumor cells, and when IFN-α-MMPS-ELP reaches the tumor site. The peptide bonds can be hydrolyzed by MMP, thereby releasing IFN-α. In mouse models of melanoma and ovarian tumor, the combined effect of ELP and MMPs significantly enhances anticancer effectiveness with fewer side effects than free IFN-α (Fig. 4B).

To treat acute myelogenous leukemia (AML), Alachkar et al. employed the FLT3 receptor as a dtarget and generated a fusion protein comprising FLT3 antibodies and ELPs. The fusion proteins displayed exceptionally persistent and prolonged therapeutic benefits in the animal and cell models of AML [51]. In the above study, CD99 was found to be highly expressed in AML, and fusion proteins of CD99 antibodies with ELPs also demonstrated beneficial therapeutic effects in the treatment of AML [52].

Tumor interleukin-1 receptor antagonist (IL-1 Ra) is used to treat several inflammatory diseases. ELP, as a fusion protein with IL-1Ra, enhances the local and systemic distribution of IL-1Ra, thereby increasing IL-1Ra efficacy and therapeutic convenience [53]. Similarly, by combining an interleukin 4 receptor (IL-4R) targeting peptide with a pro-apoptotic peptide (KLAKLAK), the bioengineered chimeric peptide AP1-ELP-KLK stimulates cellular absorption and distributes the KLAK peptide via IL-4R-mediated endocytosis. In a mouse model of glioblastoma, AP1-ELP-KLAK effectively kills glioblastoma and increases treatment efficiency [54]. Chilkoti et al. chose another death receptor agonist (DRA), the hexavalent DR5 agonist. Since ELPs form a drug reservoir in the body, a dosing frequency of up to once a week is achieved, thereby achieving long-term anti-tumor effects (Fig. 4C) [55].

MacKay et al. has developed a worm-like fusion protein (referred to as nanoworm) as an antibody-based treatment for non-Hodgkin’s lymphoma (NHL) that overcomes the clinically observed heterogeneous therapeutic outcome of B-cell NHL and drug resistance of T-cell NHL. This nanoworm can target four specific B-cell therapeutic receptors (CD19, CD20, and HLA-DR10) and T-cell receptors (CD3) in NHL. Nanoparticulate systems binds cell surface receptors via its multivalency and activated intracellular signaling to induce apoptosis and arrests the cell cycle upon CD19 aggregation [56]. With the exception of oncology, the application of ELP expanded to ophthalmic therapy. Herein, a small peptide from the mini-αB crystallin peptide (cry) displaying neuroprotective properties was fused with an ELP called SI to form CrySI. Unlike the free mini-cry, CrySI reduced retinal degeneration and prevented oxidative stress-induced cell death following a single intravitreal therapy [57].

Peptide drugs such as glucagon-like peptide 1 (GLP-1) have shown promise for the treatment of type-II diabetes, but they are degraded by dipeptidyl peptidase (DPPIV) in the body and lose their biological activity, with a half-life of about 2 min, requiring frequent administration to maintain therapeutic effects, which seriously affects patient compliance [92]. Chilkoti et al. produced a fusion protein of GLP1 with ELPs to address the short half-life of the endogenous ligand of the GLP1 receptor caused by enzymatic inactivation and rapid elimination. This protein could form a subcutaneous reservoir following a single injection and released with zero-grade kinetics with a cycling cycle of up to ten days or more. [29]. The fibroblast growth factor 21 (FGF-21) can improve insulin sensitivity and insulin resistance and can be used in type II diabetes. However, because FGF-21 is easily and rapidly cleared in the organism, its therapeutic effect is often unsatisfactory [93]. Thus, FGF-21 fused with ELP was recombinantly expressed to enhance the therapeutic use of the protein [58]. Once administered subcutaneously, ELP-FGF-21 can maintain the blood sugar levels of obese mice for up to 5 days. Correspondingly, the ELP fusion provided a controlled release mechanism that decreased injection frequency and enhanced the pharmacological efficacy of FGF-21 as a protein medication for treating metabolic disorders [59].

Studies have shown that keratinocyte growth factor (KGF) has an anti-apoptotic effect on epithelial cells [94]. Although topically applied KGF is ineffective, KGF delivered through nanocarriers [95] can accelerate wound healing, which shows that appropriate delivery methods are beneficial to improving the efficacy of KGF. Yarmush et al. fused the cytoprotective peptide ARA290 with ELP to generate KGF-ELP and ARA290-ELP fusion proteins, KGF-ELP stimulates the proliferation and migration of keratin-forming cells, while ARA290-ELP protects cells from apoptosis. Their combination can promote wound healing without eliciting an immune response, thereby prolonging the activity of the therapeutic molecule at the site of injury [60]. One cause of diabetic damage is the nonenzymatic glycosylation of proteins caused by hyperglycemia and the formation of advanced glycosylation end products (AGEs). When AGEs combine with its receptor (RAGE), proinflammatory signals are generated, which can inhibit wound healing. The soluble form of RAGE (sRAGE) can competitively inhibit AGE-mediated signaling. In diabetic mice, vRAGE-ELP decreased AGE and associated proinflammatory markers to 62% and 85%, respectively, in addition to enhancing wound closure; conversely, AGE and associated proinflammatory markers were reduced to 90% and 100%, respectively, in the control-treated animals [61].

In renal vascular disease, intrarenal administration of VEGF can stimulate microvascular repair and improve microvascular density, which suggests that using vascular endothelial growth factor (VEGF) to stimulate renal microvascular repair may be a feasible strategy for chronic kidney disease (CKD) [96]. Bidwell III et al. fused VEGF with ELP and designed a therapeutic material to gradually restore renal microvessels through ELP–VEGF treatment. Renal microvascular density and fibrosis were also improved, reducing the expression of inflammatory mediators. The key factors behind the renal recovery with ELP–VEGF treatment was the VEGF signaling restoration, the conversion of renal macrophages to the VEGF-expressing M2 phenotype, and the sustained restoration of renal function and microvascular integrity in CKD [62]. However, in future studies, the therapeutic concentration in vivo needs to be optimized to achieve the best therapeutic effect. In addition, targeting remains a key point to overcome for tumors such as brain tumors due to the composition of fusion peptides.

### Drug delivery

In cancer therapy, drug delivery focuses primarily on delivering an adequate amount of medication to the disease location, thereby minimizing drug dispersion in healthy tissue [97, 98]. In order to do this, two basic tactics that both aim to change the drug’s pharmacokinetic characteristics have been widely used. Using their inherent physicochemical features, well-defined nanoparticles are a frequently utilized delivery system that can encapsulate drugs and regulate their biodistribution [39, 99]. According to some studies, the temperature of the tumor site is higher than the average body temperature, indicating that it may function as an endogenous stimulant for drug release [100]. In terms of critical temperature, the LCST of ELP nanocarriers can be adjusted to 37 °C [101], and ought to controllably discharge its polymer payload [102]. The second is the addition of a tiny component to the medication through covalent modification, which provides acceptable pharmacokinetic characteristics while temporarily masking or limiting the drug’s bioactivity. ELPs may be precisely controlled in terms of shape and chemistry to obtain drug binding sites, as they are genetically encoded and recombinantly expressed. Accordingly, ELPs are widely used in the field of designing targeted biomolecules and in drug transport [74, 103].

#### Nanocarrier

Nanocarriers have several advantages, including a high drug-loading capacity, the ability to evade phagocytosis by the reticuloendothelial system, slower renal clearance, and prolonged drug circulation duration in the body [104–107]. ELPs can form a series of nanoparticles through self-assembly, and the peptides themselves also have good biocompatibility, which makes ELPs have great potential as drug carriers and have been used to deliver anti-cancer drugs (Table 2) [108]. When injected nanoparticles are in direct contact with tumor tissue, the effectiveness and safety of cancer treatment have increased [109]. ELP nanovesicles have been used to load hydrophobic drugs in their alkyl nuclei, while the shells have been changed to enhance intracellular delivery [26]. Additionally, modifications were implemented to increase targeting accuracy and intracellular drug delivery efficiency [110].

**Table 2:** ELPs as nanocarriers discussed in this review

Carrier composition	Drug loading mechanism	Tt (℃)	Half-life (h)	Loaded drug	Size(nm) /37℃	Treatment effect	Ref
M-ELP_90A,120_ M-ELP_90A,80_	Passive diffusion	28.8 ~ 60.4	2.34 ± 0.50	Doxorubicin paclitaxel	22.7 ± 0.6	Exhibit cytotoxic effects on cancer cells, Prolong blood circulation time.	[26]
FKBP12-ELP-RGD	Affinity between FKBP12/Rapa	25.0		Dapamycin (Rapa)	22.7 ± 0.2	Inhibit the mTOR signaling pathway, Reduce hemolytic side effects, hepatotoxicity and nephrotoxicity of the drug.	[112]
Cyclophilin A (CypA)-ELP(A192)	Affinity between CypA/CsA	49.3	957.3	Cyclosporine A (CsA)	a.7.4 ± 0.7 b. 113.0 ± 59.1	Prolong the drug half-life, Increase the mean residence time, Reduce renal drug toxicity.	[113]
ELP(E)_120_	Cysteine disulfide coupling		18.2	Paclitaxel	R_h_:58 ± 0.2 R_g_:49 ± 0.1	Prolong drug cycle time, A single dose of treatment almost completely eradicated the tumor.	[114]
F-TRAP ELP	C-terminal binding domain attachment of F-TRAP	35		Fluorine-19 (^19^ F)	30.3 ± 0.6	F-TRAP signal can be detected within 7 min, Effective treatment of MCF-7 breast adenocarcinoma cells.	[115]

Compared to the disadvantages associated with synthetic polymer drug carriers, such as high polydispersity, poor encapsulation stability, and limited biodegradability [111], ELPs have superior monodispersity, biodegradability and biocompatibility, in preclinical and clinical trials, various ELP self-assembled peptide structures have been utilized to load anticancer medications such doxorubicin (Dox), paclitaxel, and rapamycin. By binding ELP to drug molecule receptors, novel biocompatible and drug-loading-efficient peptide-drug carriers have been generated by various researchers [30, 109]. MacKay et al. reported an ELP nanoparticle with non-covalent drug binding, high affinity, and integrin-mediated cellular uptake. The ELP-Rapa nanoparticles did not cause hemolysis, interfere with platelet or plasma coagulation activity, or activate complement. For the first time, ELP-Rapa was proven to effectively inhibit the mTOR signaling pathway in mice of HR + breast cancer. The drug-induced hemolytic side effects, hepatotoxicity, and nephrotoxicity were attenuated [112]. The human cyclosporine A (CsA) receptor sequence has also been fused with ELPs to design a novel CsA drug carrier that enhances CsA loading and therapeutic safety. Unlike conventional drug encapsulation methods, this new method is surfactant-free and does not involve the breaking of covalent bonds; instead, it relies on highly specific binding between the drug and its associated receptor protein. The vector blocked the NFAT/Calcineurin/IL-2 pathway, increased the drug’s in vivo retention period, and significantly decreased its nephrotoxicity [113].

ELPs can self-assemble into tunable nanostructures that can be employed for the physical encapsulation and delivery of hydrophobic small-molecule anticancer drugs. Chilkoti et al. fused the yeast N-myristoyltransferase sequence with the ELP sequence, and myristic acid modification increased the ELP production, the reversible phase behavior of the ELP is maintained after myristoylation, and it may be tuned to operate at temperatures ranging between 30 ℃ and 60 ℃. Furthermore, myristoylated ELPs provide a diverse substrate for self-assembly into micelles of various sizes and shapes formed via genetic preprogramming. Hydrophobic small-molecule drugs, such as Dox and paclitaxel, are cytotoxic to 4T1 and PC3-luc cells due to their passive diffusion into the lipid core, their plasma circulation time was longer than the free drug, and the potency was comparable to chemically conjugated analogs [26].

To improve drug availability and improve the pharmacokinetics of small-molecule drugs, zwitterionic peptides (ZIPPs) have been developed to prevent absorption by the reticuloendothelial system, when paclitaxel was coupled to ZIPP, it self-assembles into a stable micelle structure due to the amphiphilic nature of the polypeptide chain at this point. This carrier-loaded paclitaxel has a 17-fold longer half-life than free paclitaxel. The administration of a single dosage of ZIPP paclitaxel nanoparticles to animals with extremely aggressive prostate or colon cancer can nearly completely eradicate the tumor, compared to free Paclitaxel, these nanoparticles have a longer blood circulation time and a longer duration of therapeutic effect [114].

Champion et al. incorporated pAzF into ZR–ELP vesicles, under UV irradiation, the addition of pAzF crosslinked the vesicles and increased their stability. Moreover, water-soluble Dox can be easily encapsulated by mixing with protein amphiphiles, followed by a thermally triggered phase transition. Compared to unmodified ELP, Tt and micelle sizes of ELP–Dox conjugates are significantly decreased. Dox-laden vesicles are internalized in HeLa cells and release cytotoxic Dox. Replacing the guest residues of ELP with more hydrophobic amino acids will decrease the extension of the ELP chain, resulting in a slower release of Dox. An increase in the hydrophobicity of ELP could potentially improve encapsulation efficiency and permit the binding of less hydrophobic drugs [116].

**Fig. 5:**
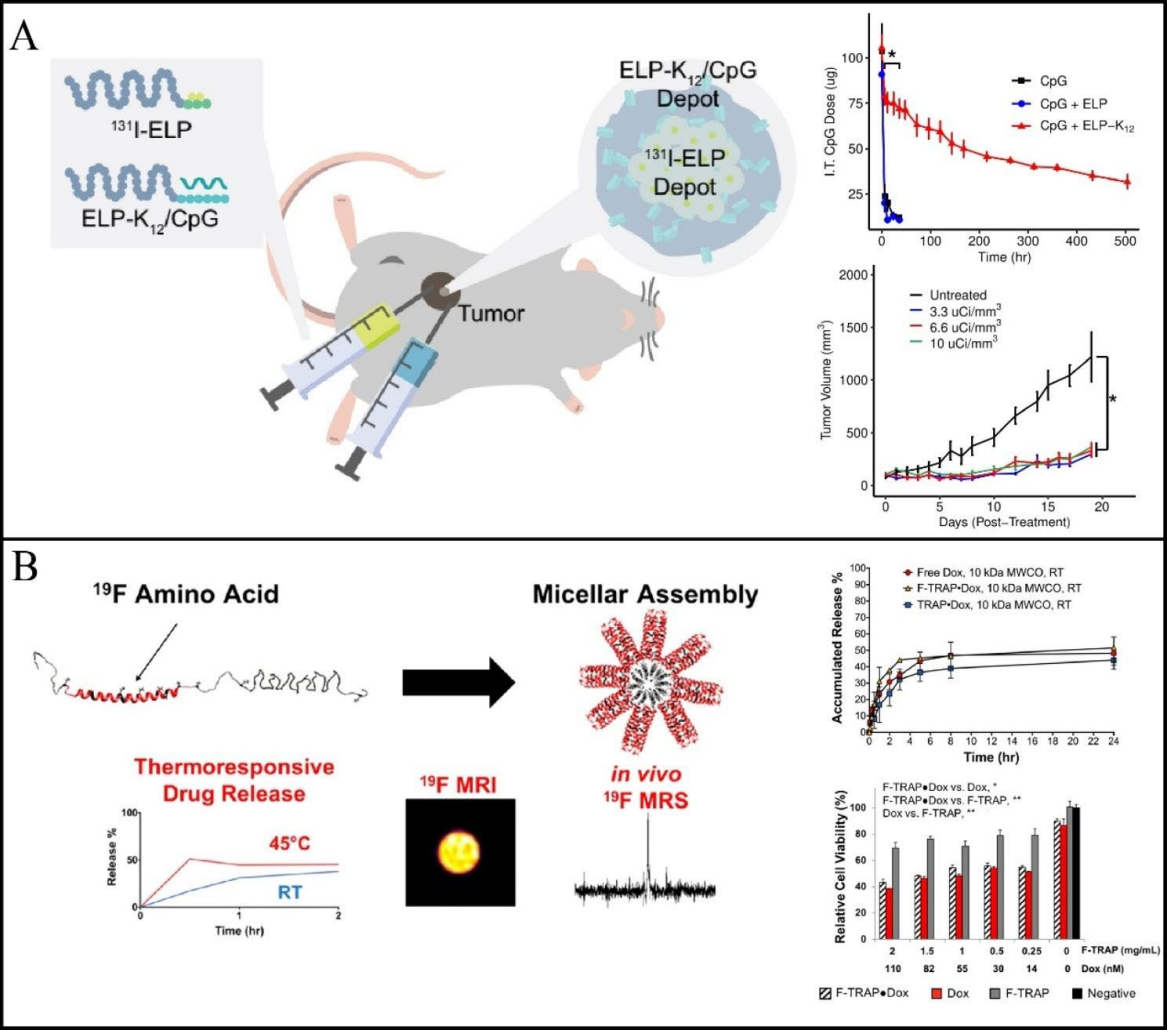
** A** Schematic of combination of ^131^I-ELP brachytherapy and ELP-K12/CpG immunotherapy. I.T. retention of CpG delivered with or without an ELP depot over time. Compared with the control group, ^131^I-ELP effectively inhibited tumor growth. (Reproduced with permission from Ref. [117] Copyright 2022, Elsevier B.V.) **B** Schematic representation of F-TRAP assembly, drug release by ^19^ F MRS and zero-echo time (ZTE) for ^19^ F MRI. Both F-TRAP-Dox or TRAP-Dox can achieve drug release similar to that of free Dox. Cell viability assay for F-TRAP-Dox, Dox and F-TRAP at 37 °C. (Reproduced with permission from Ref. [115] Copyright 2019, American Chemical Society)

Chilkoti and colleagues developed an ELP-based reservoir that prolonged intratumoral retention for over 21 days, effectively delivering CpG oligonucleotide immunostimulant. This approach increased cellular uptake of CpG, leading to slowed primary tumor growth and reduced lung metastasis. Furthermore, the combination of ^131^I-ELP brachytherapy and CpG administration effectively inhibited the growth of 4T1 tumors. (Fig. 5A) [117].

Montclar et al. developed the biosynthesis of a protein block copolymer named “fluorinated thermally responsive assembly protein” (F-TRAP), which assembled into a monodisperse structure to enhance the functionality of ELP nanocarriers. These nanomicelles, which have intriguing ^19^ F NMR properties, can encapsulate and release small medicinal compounds, making them potentially valuable as diagnostic and therapeutic agents. In addition, the small-molecule chemotherapy drug, Dox, can also be encapsulated by this fluorine-containing micelle, which releases its load in a thermoresponsive manner, and the therapeutic effect of F–TRAP–Dox can be enhanced under high-temperature conditions, providing an intriguing avenue for developing thermally responsive ^19^ F MRI/MRS-traceable therapeutics (Fig. 5B) [115].

#### Peptide-drug conjugates

In some cases, materials can be designed to form prodrugs with drugs, reducing the drug’s toxic side effects before reaching the disease site. Upon reaching the disease site and under specific conditions (such as pH or enzymes), the material can release the drug, alter pharmacokinetics, improve bioavailability, and enhance drug efficacy [118, 119]. The prodrug strategy lowers early drug release and reduces the load of carrier materials on the body when compared to nanodrug carrier approaches. Both approaches can reduce the patient’s metabolic load, thereby improving the therapeutic outcome. Drug, linker, and peptide are the three primary components of peptide-drug conjugates (PDCs) [118]. Since ELP is produced and developed through genetic engineering technology, it has the characteristics of adding hydrophobic amino acids in the sequence to adjust the hydrophobicity and Tt of ELPs, and adding charged amino acids to make the surface carry charges. The hydrophobicity can be adjusted according to the characteristics of different drugs. and charge amount, are potential choices for forming a PDC [119]. The incorporation of ELPs can confer PDCs with extensive functionality as amino acid sequences can be chosen to modify the physicochemical features of the conjugates and enable active targeting of specific receptors on the tumor cell surface. PDCs composed of ELPs, which are typically biodegradable, do not elicit undesired immunogenic responses (Table 3).

**Table 3:** ELPs used to form peptide-drug conjugates discussed in this review

ELPs sequences	Half-life (h)	Size (nm)	Linker	Treatment effect	Ref
(VPGVG)_80_(VPGSG)_60_	-	R_h_:41.8 R_g_:37.2	Telechelic hydroxylamine	Targeted toxicity to multiple cancer cell lines	[91]
(XGVPG)_160_ X = V: G: A = 1:7:8	4.2 ± 1.34	R_h_:30.1 ± 10.4 R_g_:81.5 ± 5.8	(GGC)_8_	Increase NIC tumor accumulation, Enhance antitumor activity	[120]
(VPGAG)_160_	29.4 ± 0.8	40–50	Albumin-binding domain (ABD)	Nanoscale affinity for human and mouse albumin Improved pharmacokinetic profile Prolonge intratumoral retention time	[121]
(AGVPG)_160_	12.8 ± 2.2	R_h_:56 R_g_:57	(YG)_6_(CGG)_8_	Plasma half-life prolongation Increased tumor accumulation Tumor suppressor effect is better than free drug	[122]

Chilkoti et al. developed artificial recombinant chimeric polypeptides (CPs) containing (Gly-Gly-Cys)_8_ [41], CPs were coupled with Niclosamide (NIC), and 100 nm monodisperse nanoparticles were spontaneously generated from the CP-NIC coupling. Compared to free Niclosamide, CP-NIC nanoparticles administered intravenously considerably increased NIC exposure [120].

To enhance targeting and prolong the drug cycle to deliver drugs to tumors, Chilkoti et al. developed an albumin-binding peptide conjugate that binds exclusively to endogenous albumin and takes advantage of its pharmacokinetics and pharmacodynamics, a pH-sensitive linker couples Dox with an albumin-binding domain (ABD). ABD–Dox remains water soluble following conjugation with Dox, and experiments with mice have demonstrated that ABD-Dox exhibited nanomolar affinity for both human and mouse albumin and the plasma half-life reached 29.4 h. After 2 h of injection, the intratumoral concentration of ABD–Dox remains high. However, ABD–Dox remains concentrated in the tumor for at least 72 h, where it accumulates 120 times more than free Dox at that time [121].

The Cys residue of an asymmetric triblock polypeptide (ATBP) can be covalently linked to maleimide derivatives, such as gemcitabine (GEM). All of these chemicals, including ATBP, were capable of self-assembling into nanoparticles. Experiments on mice with colon cancer revealed that ATBP–GEM caused considerable tumor regression compared to the same dose of free drug, numerous tumor cell lines exhibit high tumor cell death when exposed to ATBP–GEM conjugated nanoparticles [122]. Chilkoti et al. utilized the bio-orthologous residue pacetyl phenylalanine, which was included in the sequence of ELPs, as a drug attachment site with coupled Adriamycin to investigate various drug binding modalities, EgA1 nanobodies target human epidermal growth factor receptors, and these nanoparticles are more cytotoxic than nontargeting carriers [91].

To better control the release of drugs within the tumor, overcome multidrug resistance induced by chemotherapeutic drugs, and enhance the effectiveness of tumor treatment, Raucher et al. developed an ELP system with enzyme linkers, this system includes a cell-penetrating peptide (CPP), ELP, matrix metalloproteinase (MMP) substrate, and 6-maleimidocaproyl amide derivative of Dox. This approach achieves four times the cell penetration of the ELP-Dox combination and causes the death of a large number of breast cancer cells. The CPP complex Dox released from ELP was more effective than free Dox in killing Dox-resistant tumors [123]. A pH-sensitive linker between ELP and the drug allows the drug to be released under low pH conditions, and the structure still has a killing impact on Dox-resistant tumor cells, enabling a higher accumulation of drugs at the tumor site and a longer plasma half-life [124].

### ELPs hydrogels for tissue repair

Biomolecules are the finest biomaterial for the production of hydrogels because they are biomimetic and adaptable. Hydrogels have a unique network structure that provides a three-dimensional (3D) environment with exceptionally high hydrophilicity, biocompatibility, and soft physical properties of biological tissues, thereby mimicking the neural niche. However, ELPs have adaptive physical properties and biocompatibility to produce hydrogels with tunable qualities, such as adjustable Tt, high tensile strength, and drug storage capacity, making them a suitable material for tissue engineering [125]. Correspondingly, ELP hydrogels can provide a 3D bioactive interface that enables 3D culture, thereby demonstrating their significant potential for the repair of cartilage repair and microvascular regeneration as well as for tissue engineering applications [46, 126, 127] (Table 4).

**Table 4:** ELPs hydrogels for tissue repair discussed in this review

Hydrogels composition	Tt/℃	Coss-linking principle	Bioactive part	Result	Ref
ELP-HYD	25	Dynamic covalent hydrazone bonds	-	Cultured cells maintain the ability to differentiate into multiple lineages.	[133]
ELP-SELR	24.6	-	L-Arg-Gly-L-Asp (RGD)	Encapsulate cells, maintain cell viability and cell proliferation ability	[136]
ELP-HA-SpyTag-TriCatcher	-	Spontaneous amidation	Protein partner (SpyCatcher)	Fibroblasts and breast epithelial cells can grow, separate, and spread.	[137]
YIGSR/RGD-ELP	-	-	Cell-adhesive domains RGD cell-adhesive ligand YIGSR	Increase matrix hardness, Increase adipogenic and osteogenic differentiation markers.	[145]
ELP, SDBS, DEAB, DDAB	37	Supramolecular assembly	L-Arg-Gly-L-Asp (RGD)	Acumulation of intracellular CaP, Promote osteogenic differentiation of BMSCs	[147]
K_72_-ELP, CS, PEG-COO^−^	-	Amino groups interact with carboxylate and sulfonate groups	Chondroitin sulfate	Repair damaged cartilage, Enhance horizontal integration of tissues.	[148]
Silk-ELP,	60	Dehydrothermal	ELP	Support the adhesion, proliferation and differentiation of BMSCs or chondrocytes, Enhance cartilage tissue repair	[149]
ELP, Collagen gel	25	Hydrogen bond, hydrophobic interactions	Cell-binding domains RGD enzymatically degradable sequences (GTAR)	Produce cellularized tubular structures, Reproduce mechanical behavior of vascular ECM.	[150]
ELPs, PEG, IKVAV		Thiol-ene photo-cross-linking reactions	Adhesion peptide IKVAV	No obvious inflammation, Induce the formation of blood vessels and nerve endings in tissues.	[151]

#### 3D cell culture

2D cultured cells, various complicated biological functions such as cell invasion, cell death, transcriptional regulation, and receptor expression are compelled to change, along with cell proliferation and antiapoptosis [128]. As a result, researchers are constantly developing and supplying novel in vitro 3D cell culture platforms to help researchers to circumvent these limitations. Compared to the conventional 2D cellular approach, 3D culture technologies have improved in vitro culture techniques to better imitate the physiological environment. The primary objective of 3D culture is to imitate the ECM structure of tissues [129]. As a recombinant protein engineering material, ELP overcomes some shortcomings of conventional chemically synthesized polymer materials, such as difficulty in determining initial mechanical properties, poor cell adhesion, and difficulty in biodegradation. Before ELP production, based on the sequence structure characteristics of ELP, the biomaterials of the ELP can be directly obtained by researchers in order to meet anticipated demand [130, 131].

Due to the restricted ability of human corneal endothelial cells to divide in vivo and the lack of corneal donors, bioengineering of the corneal endothelium (CE) using replacement cells is gaining popularity. Alizadeh et al. spliced arginylglycylaspartic acid (RGD) functionalized ELP onto G4 dendritic macromolecules called elastin-mimetic dendritic macromolecules (EMD). After human adipose mesenchymal stem cells were transformed into neurospheres on a small-molecule-induced poly-L-lysine, cell sheets, including cell connections, were obtained by lowering the temperature. Thereafter, neural crest cells underwent differentiation into CE-like cells at EMD [132].

To investigate the potential of ELP as a cell culture matrix, Heilshorn et al. developed injectable hydrogels by simply mixing ELP and aldehyde-modified hyaluronic acid. The cultured cells were uniformly retained in a 3D environment and cultured for 3 weeks after injection; the encapsulated cells maintained their ability to differentiate into multiple lineages, including chondrocytes, adipocytes, and osteoblast types [133]. As a substrate used for cell culture, the degradability and stiffness of the substrate can have an impact on cell proliferation and differentiation. Additionally, they found that neural progenitor cells (NPCs) maintained stem cell factor expression with high-degradability gels with elevated levels of stem cells associated with the activating histone marker H3K4me3. By comparison, in gels with poor degradation properties, NPCs did not proliferate or senesce, exhibited tighter, rounded nuclei with heterochromatin in the periphery, and maintained expression of potentially therapeutically relevant neurotrophic factors [134]. By regulating hydrogel stiffness using ELP hydrophilicity, encapsulated hMSC, HUVEC and hNPC exhibited different cell type-dependent responses in cell morphology and spreading [135].

Silk-elastin-like recombinamers (SELRs) are typically created by combining ELP and silk-like polypeptides. When hMSCs were cultured with SELR hydrogels, the cell number increased uniformly until full confluence was reached at 30 days, indicating that the cells could proliferate [136]. The cells were observed to attach well to the pore walls under fluorescence microscopy, either singly or in small groups, suggesting that these hydrogels can affect cell behavior. Howarth et al. used hyaluronic acid (HA)–SpyTag and TriCatcher to build a rapid hydrogel through spontaneous amidation, high-viability fibroblasts could be cultured in the hydrogel, and fibronectin impacted their dissemination. The adhesion of Epithelial cell adhesion molecule (EpCAM) to the hydrogel caused mammary epithelial cells to dissociate and disseminate [137]. The regulation of cell adhesion and cell diffusion by 3D cell culture matrix formed by ELP often depends on the addition of functional peptides such as RGD peptide. In the future, it is necessary to further develop the function of ELP itself for cell growth and differentiation.

#### Cartilage repair

The avascular cartilage in the joints has a low capacity to heal after damage or inflammation [138]. Cartilage lesions not only impose significant financial and mental difficulties on patients but may also increase the risk of joint deterioration, deformity, and disability in severe cases. By modifying the bioscaffold material, cell adhesion can be achieved and tissue growth can be promoted to restore bone tissue such as cartilage. The increase in type-II collagen and sulfated glycans demonstrated that ELPs can promote the development of mesenchymal stem cells (MSCs) or adipose tissue–derived stem cells (ADSCs) toward a chondrogenic lineage without the inclusion of chondrocyte-specific growth factors [12].

However, the mechanical strength of non-crosslinked ELP aggregates is significantly lower than the dynamic shear stiffness of cartilage [139]. Therefore, the development of crosslinked ELP is necessary to enhance its dynamic shear stiffness. In a study by the Yee team [140], genipin was employed to crosslink ELP, resulting in the formation of a robust hydrogel. After six weeks of implantation in damaged rabbit cartilage, the hydrogel exhibited an aggregate modulus similar to that of native articular cartilage, without adversely affecting new cartilage formation. Olsen et al. [141] employed long-range helical oxidation to extend ELP chains, leading to the consecutive crosslinking of Cysteine-flanked ELPs under oxidative conditions. This method augmented the average molecular weight of the ELP component, resulting in coacervates with increased entanglements. Consequently, it promoted physical crosslinking, enhancing gel stiffness, and sustaining cell growth for a duration of one week.

The stiffness of the scaffold material also influences lineage development in bone-derived mesenchymal stem cells (BMSCs) [142]. Numerous studies have demonstrated that on a relatively soft stroma, MSCs differentiate into adipogenic lineages, whereas on a stiffer stroma, they differentiate into osteogenic lineages [143, 144]. Recombinant ELPs are ideal for studying the response of MSCs to matrix stiffness in a macroporous environment. Thus, MSCs that have been grown within macroporous ELPs containing either RGD or YIGSR cell-adhesive ligands as substrates responded to increasing substrate stiffness by an increase in lipogenic and osteogenic differentiation markers [145]. Yang et al. created ELP-HA hydrogels using dynamic hydrazone bonds. As the proportion of HA in the hydrogel increased, the hardness remained constant. Increasing the HA concentration led to a dose-dependent increase in the expression of cartilage-specific genes and enhanced the deposition of sulfated glycosaminoglycans, facilitating cartilage regeneration [146].

Liu et al. fabricated a series of structured organogel–derived protein fibers (KELP) for cellular mechanobiology studies using highly charged ELPs and counterionic surfactants. The insertion of RGD tripeptide facilitated cell adhesion to the surface of the fiber. After 2 days of culturing, the majority of HeLa cells were still viable, with a survival rate of over 90%. BMSCs were subsequently inoculated on KELP fibers, and on Day 1, most cells were found to be spherical. BMSCs started adhering to the surface of organic fibers after 1 week of incubation. On Day 14, the number of cells connected to the fibers continued to increase and the cells expanded in the direction of the fibers. Biomineralization assays revealed intracellular accumulation of calcium.

The presence of CaP in the KELP experimental group was demonstrated using silver staining. The feasibility of ELP organogel application for the osteogenic differentiation of BMSC was demonstrated [147].

Typically, the addition of signaling molecules, such as transforming growth factor, to gel scaffolds results in their fast release, thus limiting their long-term therapeutic effects. In contrast, the repair effect on bone tissue can be effectively enhanced by forming a gel structure with substances that promote bone tissue repair. Adding chondroitin sulfate to ELP improves cartilage development, and ECM synthesis [148]. Silk fibroin (SF) promotes cartilage repair and is biocompatible and biodegradable [152]. Chen et al. designed S–ELP–DHT hydrogel with a scaffold improved by fusing silk-fiber with ELP via dehydration heat treatment. The S–ELP–DHT scaffold promotes BMSC and chondrocyte adhesion, proliferation, and differentiation in vitro as well as repair of subchondral bone and cartilage [149]. Atsttrin is an engineered chondroprotective derivative of an anti-inflammatory growth factor, pregranular protein. Liu et al. combined ELP and cartilage oligomeric matrix protein into a hydrogel and then combined it with Atsttrin to prolong the release of Atsttrin, inhibit chondrocyte catabolism, and promote anabolic signaling in vitro to prevent the onset and progression of post-traumatic osteoarthritis effectively [153]. Cartilage repair is subject to the characteristics of materials, adding rigid materials such as bioglass into ELP is conducive to bone tissue repair, but non-biological materials have poor biocompatibility. At the same time, bone tissue repair requires materials to promote cell adhesion, osteogenic differentiation and other functions, and these functions of ELP are yet to be developed.

#### Microvascular regeneration

Vascular regeneration is particularly crucial for tissue engineering projects when developing huge complicated tissues or even human organs using tissue engineering. Furthermore, microvascular generation has been demonstrated to be pivotal in metastasis, tissue regeneration, and wound healing [154]. In recent years, ELP hydrogels have become a popular research topic for Microvascular generation owing to their biocompatibility, biodegradability, ease of handling, and rapid wound closure activity [155, 156].

By using ELPs to simulate the extracellular matrix and regulate signaling between cells and the extracellular matrix, it will contribute to research on microvascular regeneration and tissue engineering. Girotti et al. introduced cell adhesion peptide RGD and Arg-Glu-Asp-Val (REDV) peptide into ELPs, the adhesion and spreading of endothelial cells on the scaffold were found to be considerably higher than that of fibroblasts. Similar to natural elastin, ELP is enzymatically hydrolyzed to produce elastin-derived peptides or matrix factors, which may regulate important cellular activities [157]. Microvascular regeneration is essential for the effective repair of various tissues and is one of the major obstacles to tissue regeneration. José et al. designed a tubular structure composed of a coaxial binary ELP complex with a crosslinked structural domain and a conjugated VEGF–derived peptide (QK) for promoting blood vessel regeneration that is responsive to urokinase and fibrinogen activator proteases, this study demonstrates the ability of ELPs nanostructures to rapidly regenerate blood vessels and guide cell infiltration and capillary formation due to the presence of proteolytic sequences and QK peptides [158]. Components of ECM modulate the ischemic microenvironment and alleviate the myocardial ischemia–induced necrosis of cardiomyocytes. Pandit et al. used two ELP systems crosslinked into hydrogels for mimicking ECM [159]. The resulting recombinant elastin hydrogel is sensitive to cleavage by overexpressed matrix metalloproteinases and can be efficiently degraded in vivo. Compared with untreated animals, the ischemic core area treated with ELR hydrogel had less fibrosis, more microangiogenesis, improved the ischemic microenvironment, and increased the number of GATA4 cardiomyocytes [160].

Extreme limb ischemia (CLI) is a symptom of severe peripheral artery disease. In this regard, promoting angioplasty is a beneficial CLI treatment method. According to Pandit et al., ELP hydrogels can regulate crucial angiogenic signaling pathways to promote capillary growth and ECM remodeling [161]. Accordingly, MSCs were added to ELP hydrogel to improve the therapeutic effect on limb ischemia. This substantially reduced inflammation at the injection site, considerably improved tissue reperfusion, and increased vascular density following injection [162]. In addition, Chilkoti et al. created a series of partially ordered polypeptides using recombinant sequence design to investigate emerging hierarchical topologies, these materials retain the thermally sensitive features of ELPs, with variable phase transition temperatures and the capacity to build reversibly porous, viscoelastic, and stable network architectures above the phase transition temperature. When entering the body, they can rapidly integrate into the surrounding tissues with minimal inflammation and high vascularization [163].

Selective adhesion of endothelial cells (EC) to biomaterials contributes to microvascular regeneration, and the capillary network generated by EC adhesion and migration is essential for tissue regeneration [164]. However, when the anticoagulant activity of EC is low, it is easy to cause blood coagulation around the material. Uygun et al. enhanced reendothelialization of decellularized rat liver scaffolds by enhancing endothelial cell adhesion to the vascular wall surface by binding to the cell-binding domain of REDV (arginine–glutamate–aspartate–valine (Arg-Glu-Asp-Val)), REDV–ELPs enhanced endothelial cell adhesion, proliferation, and spreading while substantially reducing platelet adhesion and activation [165]. Mantovani coupled the recombinant ELP with collagen gel and cells to form cellularized tubular structures. When 30% ELP/collagen (mass ratio) is used to make cellularized tubular gels, cellular activity is maintained without altering fracture strain [150]. Santos et al. developed a cell and growth factor free hydrogel composed of ELPs, PEG, and increased concentrations of the adhesion peptide IKVAV, which is capable of promoting angiogenesis and innervation. These hydrogels, implanted subcutaneously in mice, elicited no substantial inflammatory responses; however, the 50% IKVAV composition generated an increase in vessel density and the development of nervous terminations in peripheral tissue [151].

Due to the weak therapeutic effect of some drugs/cytokines, ELPs used in tissue repair are often limited by the drug dose that is loaded. At the same time, the therapeutic efficacy of pure ELP on tissue repair needs further study.

## Conclusions

This article first introduces the more hydrophobic the guest residue (X) in the classic Val-Pro-Gly-X-Gly pentapeptide sequence of ELPs, the lower the Tt. Secondly, the construction of ELPs is introduced. ELPs are mainly produced by E. coli. Since Urry first produced ELP by E. coli in 1992, the preparation methods of ELP include RDL, PRe-RDL, and OERCA.

Finally, this article provides an overview of the applications of ELPs in drug delivery and tissue repair. Due to their biocompatibility, low immunogenicity, and biodegradability, ELPs are widely used in drug delivery systems. ELP fusion proteins can be directly obtained through genetic engineering technology. Covalent conjugation of ELPs to small molecule drugs can be achieved by genetically engineering site-specific conjugation reactive residues. Through the peptide drug conjugate, the drug reservoir formed after entering the body extends the half-life of the drug. According to the phase change characteristics of ELPs, ELPs can be used for tissue repair. By modifying the ELPs peptide chain such as SpyTag and SpyCatchr, ELP can be cross-linked into a more stable gel. The addition of cell adhesion sites in the ELPs peptide chain can Enable ELPs for 3D cell culture. By adding appropriate therapeutic factors, ELPs can also be used for cartilage repair and microvascular repair. In addition, due to the thermoresponsiveness of ELPs, the ITC method can be used to purify soluble proteins containing ELPs. ELPs can be used to modify nanoparticles, such as binding to gold nanoparticles through sulfhydryl groups [17, 166], and binding to silica through the formation of ELP-silica peptide complexes [167].

Although ELPs have shown obvious advantages and great potential in drug delivery, they currently face problems that need to be solved. Through genetic engineering methods, ELP can only be fused at the C-terminus or N-terminus of the protein, and the methods of modifying the protein are relatively limited. The fusion of therapeutic proteins with ELP may affect the biological activity of the drug [50], and further optimization is needed in the future to preserve the activity of therapeutic proteins. In addition, ELPs have been widely studied in drug delivery and tissue repair, but there are fewer studies on the application of ELPs in other fields, which requires researchers to continue to study ELPs in new fields.

## Abbreviations

ELPs: Elastin-like polypeptides
ECM: extracellular matrix
LCST: lower critical solution temperature
Tt: transition temperature
ITT: inverse temperature transition
SPPS: solid-phase peptide synthesis
ITC: inverse transformation cycling
NCAAs: non-canonical amino acids
PCE: photocrosslinked ELP
PCD: photo-crosslinkable deblock
PEG: Polyethylene glycol
pAzF: p-azidophenylalanine
IFN: interferon
MMPS: matrix metalloproteinase substrate
Niclosamide: (NIC)
AML: acute myelogenous leukemia
NHL: non-Hodgkin’s lymphoma
GLP1: Glucagon-like peptide-1
FGF-21: fibroblast growth factor 21
GBM: glioblastoma
KGF: keratinocyte growth factor
AGEs: advanced glycosylation end products
RAGE: AGE receptor
EpCAM: epithelial cell adhesion molecule
VEGF: vascular endothelial growth factor
HA: hyaluronic acid
HCEC: human corneal endothelial cells
CE: corneal endothelium
HADSC: human adipose mesenchymal stem cells
NCCs: neural crest cells
NPCS: neural progenitor cells
HMSC: human mesenchymal stem cells
HUVEC: human umbilical vein endothelial cell
Hnpc: human neural progenitor cells
Sgag: sulfated glycans
ADSCs: adipose tissue derived stem cells
rMSCs: rice mesenchymal stem cells
BMSCs: bone-derived mesenchymal stem cells
TGF: transforming growth factor
CS: chondroitin sulfate
SF: Silk fibroin
DHT: dehydration heat treatment
QK: VEGF-derived peptide
HDFn: human dermal fibroblasts
EC: endothelial cells
CsA: cyclosporine A
ZIPP: zwitterionic peptide
ABD: albumin-binding domain
ATBP: asymmetric triblock polypeptide
GEM: gemcitabine
AMPs: antimicrobial peptides
Doc: Dockerin
PAA: poly (acrylic acid)
Cys: cysteine
